# The development of a collective quality system: challenges and lessons learned; a qualitative study

**DOI:** 10.1186/s12909-017-0946-8

**Published:** 2017-07-21

**Authors:** Nienke Buwalda, Jozé Braspenning, Nynke van Dijk, Mechteld Visser

**Affiliations:** 1 Department of General Practice/Family Medicine, Academic Medical Center-University of Amsterdam, Amsterdam, the Netherlands; 2 IQ Scientific Institute for Quality of Healthcare, Radboud University Medical Center, Nijmegen, the Netherlands

**Keywords:** Quality assurance, Quality system, Development, Postgraduate medical education

## Abstract

**Background:**

The ongoing professionalization of medical education means that quality systems (QSs) aimed at improving medical education also continuously have to improve*.* The aim of this paper is to describe the development of a collective QS for eight Dutch General Practitioner (GP) specialty training institutes to provide insights into the considerations that are involved in developing a QS in medical education.

**Methods:**

Experts in the field of GP education and quality assurance developed the QS. They studied the literature, prior QSs and involved stakeholders. The team interviewed the directors**,** and all meetings and steps in the development process were transcribed. All interviews and relevant documentation were analyzed. Results were checked by the developers.

**Results:**

Stakeholders agreed on the goals, the relevance of the resulting domains, and the methods to assess. However, one major theme emerged. To enable benchmarking, the team developed detailed quantifiable indicators. Especially the development of these indicators gave discussion.

**Conclusions:**

Involving stakeholders was crucial as they directed the development of the QS. The framework of the World Federation for Medical Education (WFME) provided guidance in covering all the relevant processes. The major challenge consisted of formulating indicators. Our experience indicates that the process of quantifying indicators is not straightforward. The detailed level of the indicators chosen is perhaps not always suitable for QSs in the field of medical education.

## Background

Over the past decades, there has been a growing interest in improving medical education, assuming that better education will eventually result in better doctors and therefore in better healthcare [1]. Various groups and organizations have developed guidelines for quality improvement of medical education [2]. For example, the World Federation for Medical Education (WFME) defined and formulated international standards for graduate and postgraduate medical education and for continuing professional development [3–5]. The WFME framework deals with all aspects of medical education: the organization, structure, content, process, environment, management and outcomes [2, 6].

Such guidelines provides standards that help to assess the strengths, weaknesses and needs for improvement for both institutes and their educational programs [7]. The standards assure a minimum level of quality, but they can also give rise to developments beyond the levels specified [6] and encourage institutional self-evaluation [8–11]. The standards themselves also require periodic assessment, for example with self-evaluations and peer-reviews, or a combination of these methods. The outcomes of these assessments can help develop strategic policy and planning to assure quality improvement in medical education [4].

Quality standards for a medical education institute can be defined and assessed by an external organization or by the institute itself. Usually, if an external organization formulates and assesses the standards, a national body monitors whether these standards are met: the so-called accreditation [12]. In addition to this type of external control, it can be valuable to have an internal quality system (QS). Creating such a QS includes defining standards that are geared towards improving rather than just controlling quality and choosing methods that assess these standards.

This paper provides an example of how the Dutch General Practitioner (GP) specialty training developed an internal QS. In the Netherlands, the GP specialty training is provided at eight institutes, and a national body (named RGS) is responsible for monitoring the medical specialty training in order to assess compliance with the requirements of the profession. In order to further improve the quality of their training, however, the training institutes wanted to create a structured internal QS with clear and shared criteria. In 2005, the institutes therefore started to develop their own QS (named PAUKH) that, at first, was used internally at each separate institute. By means of self-evaluation and an audit, the institutes received information about their own strengths and weaknesses.

In order to create more transparency between institutes and to the public, and because the institutes wanted to benefit more from each other, the eight Dutch GP training institutes developed a second QS (named PI). The goal was to accomplish benchmarking: the institutes scored themselves on indicators and compared the outcomes. A financial incentive was included by rewarding the best performing institutes. However, using both PAUKH and PI was time consuming, which created a strong wish to combine both systems into one structured QS. The main goal of such a new QS would be to support the GP specialty training institutes in making plans for improvement, implementing these plans, and exchanging products. Consequently, the QS should stimulate the institutes to improve their own quality and contribute to the quality of the other institutes.

While it is not always clear which standards and methods are suited to an organization [13], it is clear that the aims of a QS have to fit the aims of the institute and the standards it wishes to assess [14]. Like the international WFME standards, internal quality standards offer a framework that needs to be further elaborated and customized towards a particular local situation. The new system was named GEAR; Dutch acronym for Combined Evaluation Audit Round. The aim of this paper is to describe the development of GEAR, in order to provide insights into the considerations that are involved in developing such an internal QS in (postgraduate) medical education. In the field of medical education, there is a lack of examples of detailed QSs. In fact, as far as we know, this is the first paper that systematically reports the development of a QS. The identification and discussion of the issues that arose during its development may therefore facilitate other specialty trainings in developing a QS.

## Methods

### Context

The Netherlands has eight GP specialty training institutes. They are embedded in the departments of family medicine at the university hospitals. Together, they are responsible for approximately 700 graduates annually. The Dutch GP specialty training is a three-year postgraduate training. In their first and third year, trainees work in a GP practice under the supervision of a GP-trainer. The second year is used for clinical rotations. During the entire training period, trainees also follow a programmed curriculum at the training institute for one day a week, focusing on theoretical and practical skills.

### Data collection for this study

A project team that was commissioned by the Dutch GP specialty training was assembled to develop the new QS. This project team consisted of four GP care and quality care experts. They studied the literature and prior QSs, interviewed the directors, and discussed their outcomes with stakeholders and with each other. The first author (NB) was not involved in developing the new QS. To study the development of the QS, NB received the data from the project team. The interviews with the directors were audio recorded and anonymized. Furthermore, the project team elaborately transcribed all meetings (with the project team, directors, and the sounding board), and they described all the steps in the development process in several reports. Approximately 50 documents were studied in total.

### Data analysis

All textual data (summarized interviews and documents) in this study were explored using content analysis [15]. First, NB summarized the interviews with the directors (seven in total) and identified important text fragments by using open coding. After the coded text fragments were structured, they were subdivided into the categories ‘strengths’ or ‘weaknesses’ of both prior systems, or ‘remaining requirements’ for the new QS (potential improvements). In order to deepen the understanding of the developmental process, NB also performed a document analysis [16]. The reports of the prior QSs, minutes of meetings, memos, notes, and newsletters were read to outline the developmental process. Subsequently, themes (points of discussion) in the process were identified and studied. When additional information was needed, the members of the project team were asked for clarification. Finally, the results were checked by two members of the project team and adaptations were made when necessary.

## Results

**Fig. 1:**
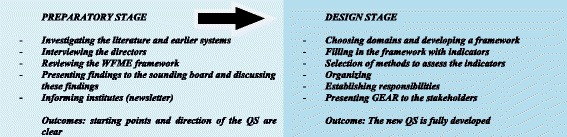
Development process GEAR

There were two stages in the development process: the preparatory stage and the design stage (Fig. 1).

### Preparatory stage

During the preparatory stage, it became clear that the literature provided no example of a detailed QS that the team could use. Therefore, the team first investigated the content, procedures, time investment and bottlenecks of the existing QSs that were in use (PAUKH and PI). It was considered important that the strengths of the prior QSs would be an integral part of the new QS (Table 1). In addition, the team interviewed the directors to ascertain their opinions about the earlier QSs and ask them to suggest improvements. The principal outcome of the interviews was that the prior QSs provided the institutes with feedback but lacked the tools for using this feedback. Therefore, the intention was that the new QS should lead to plans for improvement at each institute and to the assessment of these plans.

**Table 1:** Strengths and bottlenecks of the different quality systems that were used by the GP specialty training institutes

Prior systems	Strengths	Weaknesses
System 1: Self-evaluation + AUDIT for only internal use (PAUKH)	• In a visitation, there is personal contact with a commission and the institute • The committee can determine the atmosphere at the institute • All relevant stakeholders are involved: staff, trainees, trainers, management	• Only for internal use. Not designed to compare and exchange • The time investment is large. Preparing the institutes for a visitation takes much time. Trainees • GP-trainers, teachers and staff are required to prepare and attend • No follow-up steps after the audit. It is hard to integrate the feedback of the audit into day-to-day practice
System 2: Scoring on indicators and benchmarking (PI)	• It encourages to describe processes and policies and keep these up-to-date • Numerical outcomes facilitate comparisons between institutes	• Competition arises, because of the ranking and encourages window dressing • To support their scores, institutes have to provide much paperwork, which is complicated and time-consuming • Focuses on paper-work and not on what actually happens in practice • Only a few people are involved: the ones who collect the paperwork • No follow-up steps are planned to use the feedback into day-to-day practice

The institutes’ directors preferred to focus on transparency and a positive approach instead of encouraging competition between institutes. The new QS would therefore be designed to become an advisory tool to stimulate improvements, instead of a controlling and normative system. This would also add to the transparency as it would not encourage any ‘window-dressing’. In addition, the directors wanted the new QS to be basic, concise and up-to-date. It had to be incorporated within the current national policy and not be too complicated to work with. Finally, the collective system should facilitate the exchange of good practices between institutes (such as educational programs, local policies, and ideas to approach organizational problems), in order to allow the institutes to benefit more from each other.

In addition to studying PAUKH and PI, the team studied other QSs and accreditation frameworks for inspiration and for possible gaps in PAUKH and PI. Subsequently, the team discussed the findings with the sounding board, made up of a director of an institute, clinical trainers, a trainee, an expert in quality management and six representatives of professional associations, including a representative from the RGS (the national controlling committee). The sounding board shared the views of the directors and, in addition, advised that the QS should lead to qualitative and quantitative feedback, and that the institutes should receive support in working with the system. All stakeholders agreed that the QS should be used for at least three main objectives: quality improvement, quality assurance, and facilitating collaboration between institutes.

In this first stage, the team established the aims and starting points of the QS. To engage the institutes, they all received a progress-report newsletter.

### Design stage

1. Developing a framework
  In the design stage, the team developed a framework of domains. The domains covered the broad elements in the structure, process and outcome of the GP specialty training. The WFME framework refers to these domains as ‘areas’ [4]. First, the team chose the relevant domains of the prior QSs that would be recognizable and important for the GP specialty training institutes. For example, all stakeholders had originally agreed on the need for the further professionalization of the academic level of the institutes. For this reason, the domain ‘academic level’ was included in the new QS.
  The team carefully reviewed the WFME framework [4] to check for missing areas. For example, the WFME framework included elements that were lacking in the existing quality systems PAUKH and PI, such as facilities, learning environment and leadership. The sounding board considered these to be relevant for the GP specialty training. In addition, their advice was to pay specific attention to the learning environment in GP practice, because GPs in training spend most of their time there.
  In consultation with the stakeholders, the team eventually opted for seven domains: (1) staff; (2) management; (3) vision and quality policy; (4) academic level; (5) assessment, evaluation and results; (6) educational environment; and (7) educational program (shown in Table 2).
2. Filling in the framework with indicators
  It was considered important that the new QS should also be a benchmark tool; therefore, indicators played an important role. They had to be measurable and possess discriminative power for benchmarking. The domains were divided into sub-domains and themes, and each theme was covered by indicators.
  The stakeholders agreed that the seven domains covered the relevant processes of the GP specialty training. The team decided to first address three domains; (1) academic level; (2) assessment, evaluation and results; and (3) educational environment, as these domains were already areas of attention of the institutes, and would be the focus of the first measurement round.
  A close look at the domain ‘academic level’ (Table 3) illustrates the development process of the indicators. The opinions of various stakeholders with expertise in the domain were sought, to describe the domain from a clear perspective. This led to considerable discussion, as opinions of the stakeholders differed significantly. After the stakeholders reached consensus, a clear perspective helped to divide the domain academic level into four sub-domains: (1) educational program; (2) scientific research for the trainee; (3) staff; and (4) scientific climate.
  The sub-domains were subsequently divided into more detailed themes. The stakeholders agreed that the sub-domain educational program included two themes: (1) training in evidence based medicine (EBM) for the trainee; and (2) scientific research trainees. Ultimately, based on the themes, the team designed quantifiable indicators. To facilitate both the use of and comparisons between the indicators, it was decided to employ a uniform format in which all the indicators could be scored using the same scale with a maximum of five points. Two examples of these indicators are given in Table 3. Whenever possible, existing indicators from the two former QSs were used (such as, for example, ‘winning a scientific award’). Some of the additional indicators were derived from the national trainee survey (every two and half years, a national survey is conducted in which all trainees evaluate their own GP specialty training institute).
  There was discussion on whether the indicators truly referred to quality; they appeared to relate more to paperwork and seemed to resemble preconditions rather than quality. Furthermore, they were rigid and forced the institutes in a particular direction. For example, with the first indicator in Table 3 (education of trainees in EBM), an institute may perfectly describe its educational EBM program, including all the requirements, and score all five points, but this does not automatically imply that its program is of high quality in practice. However, all stakeholders agreed that having a good description of an educational EBM program is a relevant precondition.
  Taking this discussion into consideration, the first three domains (‘academic level’, ‘assessment, evaluation and results’ and ‘educational environment’) were covered by indicators that could be assessed, which made benchmarking possible. Finally, the team checked all indicators for accuracy and current relevance. To avoid discussion on interpretation of indicators, all domains and indicators were clearly described in a manual.
3. Selection of methods to assess the indicators
  The team selected methods to assess the indicators. The first method was self-evaluation: to obtain an overview of the current state of affairs, it was thought that the institutes would have to conduct a self-evaluation, and subsequently receive an overview of how they scored and compared to each other (benchmarking). Therefore, all institutes would have to start simultaneously by making an evaluation using a structured online questionnaire. In addition, institutes would be allowed to support their scores by uploading relevant documents.
  As the self-evaluation focuses on the indicators, a two-day audit was considered necessary to add qualitative feedback. Auditors can look beyond the indicators and paperwork and encourage the institutes to improve. Furthermore, they could explore more perspectives, such as those of trainers and trainees. There was discussion about who should be part of the audit commission. The sounding board advised the team to use external, qualified auditors who can be critical and support the institutes during the process. Furthermore, to stimulate contact and collaboration among the institutes and to create support, it was thought helpful to include a trainee, a trainer and a teacher in the commission. Consequently, the audit commission consists of two professional auditors and three representatives each from a different institute. In line with the intention to use a positive approach, the audit interview is to be carried out using the appreciative enquiry technique. This approach focuses on the positive aspects of the organization as the starting point for change. The representatives from the institutes attend a professional training day on appreciative enquiry.
  After the measurement round, the institutes will develop and implement improvement plans. The feedback enables each institute to judge from which institute they can learn, and what they can offer to the other institutes. On the basis of this information, institutes are invited to exchange good practices. To encourage this exchange, meetings are organized where institutes present their good practices to each other. Institutes can ‘shop’ at other institutes and use each other’s good practices for their improvement plans.

**Table 2:** The WFME framework and the GEAR framework

Framework WFME Postgraduate Medical Education	Framework of GEAR
1. Mission and outcomes	1. Staff
2. Training progress	2. Management
3. Assessment of Trainees	3. Vision and quality policy
4. Trainees	4. Academic level
5. Staffing	5. Assessment. Evaluation, and results
6. Training Settings and Educational Resources	6. Educational environment
7. Evaluation of Training Process	7. Educational program
8. Governance and administration	
9. Continuous Renewal

**Table 3:** Domain ‘academic level’

Sub-domains	Themes	Indicators
Educational program	Education of trainees in EBM Scientific research of trainees	1 Education of trainees in EBM* 2 Scientific research as internship for trainees* 3 Winning a scientific award
Scientific research trainees	Aiothos** Scouting ‘high potential’ students	1 Proportion of aiothos in the GP training course 2 Scouting trajectory for ‘high potential’ students
Staff	Training trainers in EBM Training teachers in EBM Use of expert teachers	1 There is a professional EBM training for the GP trainers, and they have followed the training 2 Training teachers in EBM 3 Use of expert teachers
Scientific climate	Involvement of trainees, teachers and trainers in scientific activities Involvement of scientists in education Integration of science into the daily practice	1 Involvement of trainees, teachers, and trainers in relevant scientific activities 2 Academic first-line learning/working environments 3 Involvement of scientists in the education department 4 Integration of science into the daily practice
* As an example, the first two indicators are fully described: 1 Education of trainees in EBM Training scores 1 point for each item, maximum 5 points: - Formulating a clinical question is part of the educational program and trainees make a PICO/CAT; - During the educational session or through self-study, trainees learn how and where they can find and select literature); - During the educational session, there is explicit attention for critically evaluating the literature; - Applying the evidence to the patient is demonstrably part of the educational program; − Besides specific EBM educational programs, other programs also pay attention to the EBM principles. 2 Scientific research as internship for trainees Training scores 1 point for each item, maximum 5 points: - Research internship is offered; - There are clear assessment criteria for research internship; In the past two years: - There have been at least two trainees who followed an internship or who conducted differentiation research; - At least two trainees (during research internship) held a presentation at a congress; - Internship or differentiation research has led to at least one scientific article.		
** Physician in training for general practice researcher (resulting in registration as General Practitioner and Ph.D. after 6 fulltime years of training)

**Fig. 2:**
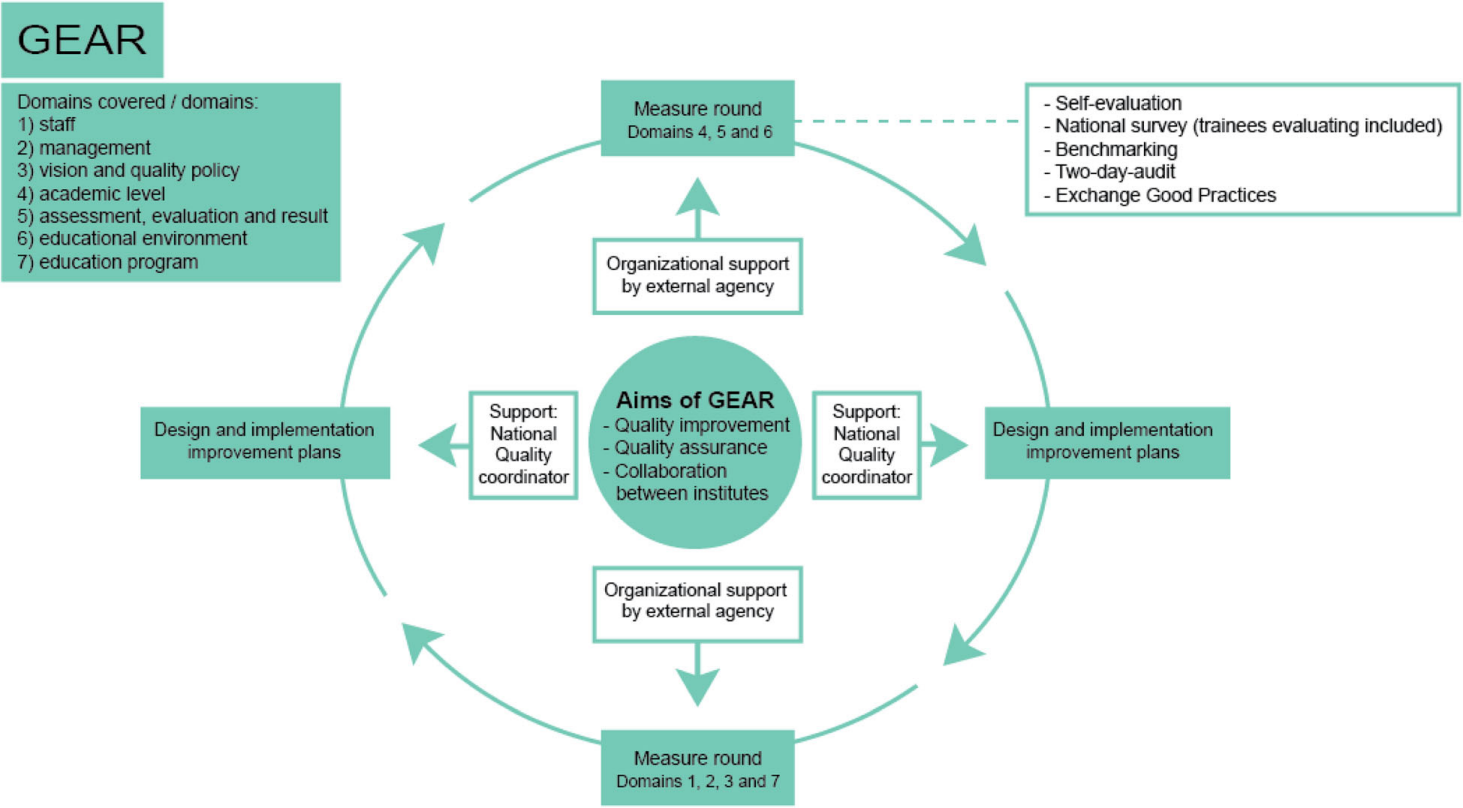
GEAR figure

4. Organizing
  An external accreditation organization was engaged to advise the team on how to organize the system. This organization would also play a role in the further implementation of the QS [17]. As the preparatory stage had made clear that the QS should not only help gather data, but that it should also enable quality improvements as a continuous process, the team further determined that the results of the improvement plans should be part of the next audit round. Because it would be too much to assess all domains at once, the team decided on two measurement rounds in five years, each of them covering half of the domains. The resulting QS of the GP specialty training institutes is shown in Fig. 2.
5. Establishing responsibilities
  The system includes national support from two sides (shown in Fig. 2). First, a national quality coordinator stimulates the institutes to exchange, collaborate and actually improve. The quality coordinator also plays an important role in the development of the system. Second, the external accreditation organization assists the institutes with the implementation of the system, and is responsible for the development of a web-based environment, for training and preparing the institutes, and for setting national deadlines.
  It was decided that a concilium, made up of representatives of the Dutch GP specialty training institutes and representatives of professional associations, would be responsible for monitoring the quality of the QS itself. This committee is advised by a special working group, consisting of experts in quality assurance, a director of one of the institutes, a clinical trainer and a trainee. This team is responsible for evaluating the QS, in order to actualize and improve the QS ‘GEAR’.

## Discussion

The main purpose of this study was to describe the development of a Quality System (QS) and to provide insight into the considerations that are involved in developing a QS in (postgraduate) medical education. Based on the literature, and in consultation with stakeholders, a project team developed this QS for the eight Dutch GP specialty training institutes. Our results indicate that we can learn some important lessons.

Formulating goals together with stakeholders at the start was an important part of the process: this gave direction concerning the design of the system. Furthermore, the collaboration encouraged the support of, and commitment from, stakeholders regarding the QS, and – as Becket and Brooks [13] point out – involving internal and external stakeholders is an important step in the development process, as it takes all different perspectives into account.

Using the World Federation for Medical Education (WFME) framework [4] proved to be another helpful step. This framework was helpful in obtaining an overview of potentially relevant processes and standards, and it served as a frame of reference. The WFME framework consists of global standards that give direction to what attributes a postgraduate medical training should have (basic standards), and it provides suggestions for further quality development. The standards were adapted somewhat to the shared local needs of the Dutch GP specialty training institutes; this adaptation to context seems necessary to allow the use of the standards in medical education that applies to that specific context [18].

The team believed benchmarking was an important element of the QS because it allows both comparison across institutes and the exchange of good practices. After the team developed a framework of domains that covered the broad elements in the structure, process and outcomes of the GP specialty training, the team had to operationalize domains into quantifiable indicators that were suitable for benchmarking. However, the transformation of quality into indicators was complex: operationalizing the broad domains into indicators caused much debate. Stakeholders argued that the indicators referred to paperwork and preconditions, and according to some of them, the indicators were too strict and left no space for other ways of organizing quality. However, most preconditions were also seen as important.

Our experience indicates that the process of quantifying indicators is not straightforward. Roffe [19] also reflects on the complexity, and the use, of numerous indicators in higher education and argues that indicators used in the industry are much more easily quantified, while indicators in higher education are more complex [20]. Measureable indicators can be scored and compared, while descriptive indicators can be explained but not be scored. Moreover, if the QS is intended to be an advisory tool instead of a normative tool, global standards – instead of detailed quantifiable indicators – can also help institutes gain insight and improve themselves. However, using global standards does not allow benchmarking.

Instead of benchmarking, other tools may be appropriate for providing a way of detecting and sharing good practices, for example — because the institutes are spread across the country — a virtual community of practice. Communities of Practice (COP) have been described as groups of people who have a common interest, interact regularly, and learn from each other [21]. A virtual COP facilitates collaboration online, and Barnett [22] noted that a virtual community seemed to be promising in overcoming isolation and improving connectedness. Institutes could, for example, have an interactive virtual community where they can ask each other for examples, and where they can share and exchange documents.

The team opted for quantitative and qualitative assessment because they complement each other, and the literature confirms that mixed methods are more appropriate because they are complementary. To substantiate indicator scores and investigate the underlying problems and opportunities to improve training quality, an audit also would be required [13, 23]. Ivers et al. [24] suggest that an audit is interactive and participatory and widely used as a quality improvement intervention.

This qualitative study provides detailed information about developing an instrument for quality assurance and enhancement, and about the inherent complexity that is involved. However, the information was gathered by reading documents and listening to the recorded interviews between the developers and the directors of the institutes, which leaves space for interpretation. In order to minimize the risk of misinterpretation, the team of developers were approached whenever there were doubts. In addition, two team members gave feedback on drafts of this paper, and one is a co-author (JB).

## Conclusions

This qualitative study into developing a collective quality system (QS) has unveiled some important challenges and lessons. In the context of developing a QS for the Dutch GP training institutes, we found that stakeholder involvement was crucial: they directed the development of the QS and its practical application. Using the WFME framework provided guidance in covering all the relevant processes of the training. The main challenge was formulating indicators. We suggest that more research is warranted to investigate to what extent this QS will serve its purpose; however, this first requires optimal implementation of the QS into the daily practice of the institutes.

**Box 1:** Practice points

• Formulating goals together with stakeholders gives direction concerning the design of a Quality System (QS).	
• The WFME framework provides relevant building blocks for quality systems.	
• In order to use a global framework (such as the WFME framework) for quality assurance, it should be adapted to local needs and circumstances.	
• The process of quantifying indicators is not straightforward. It is questionable whether quantifiable indicators are necessary.	

## Abbreviations

GEAR: Dutch acronym for combined evaluation audit round
GP: General practitioner
QS: Quality system
WFME: World federation for medical education
